# Structural, angiogenic, and immune responses influencing myocardial regeneration: a glimpse into the crucible

**DOI:** 10.1038/s41536-024-00357-z

**Published:** 2024-04-30

**Authors:** Basil M. Baccouche, Stefan Elde, Hanjay Wang, Y. Joseph Woo

**Affiliations:** Stanford University Department of Cardiothoracic Surgery, Palo Alto, CA USA

**Keywords:** Stem-cell research, Mechanisms of disease, Stem-cell therapies

## Abstract

Complete cardiac regeneration remains an elusive therapeutic goal. Although much attention has been focused on cardiomyocyte proliferation, especially in neonatal mammals, recent investigations have unearthed mechanisms by which non-cardiomyocytes, such as endothelial cells, fibroblasts, macrophages, and other immune cells, play critical roles in modulating the regenerative capacity of the injured heart. The degree to which each of these cell types influence cardiac regeneration, however, remains incompletely understood. This review highlights the roles of these non-cardiomyocytes and their respective contributions to cardiac regeneration, with emphasis on natural heart regeneration after cardiac injury during the neonatal period.

## Introduction

Ischemic heart disease (IHD) is the leading cause of death globally^1,2^. Despite the continued optimization of medical therapies and coronary revascularization strategies, the public health burden of IHD continues to grow at an alarming rate^3–5^. Cardiac regeneration has long been sought as a potential adjunctive therapy for patients with IHD. However, while various strategies to stimulate cardiac regeneration have shown promise in animal models, initial clinical trials in human patients have demonstrated inconsistent results^6–11^. This may be due in part to the inherent challenges of basic science research in cardiac regeneration, limitations salient to any review of evidence in this field. These include but are not limited to the difficulty of invasive procedures on neonatal rodents that are physiologically fragile and still fully dependent on their mothers post-operatively until well after the regenerative window has closed, variations in technical execution of cardiac injury (such as discrepancies in the infarct size generated, the amount of tissue resected in apical resection models, or the level of LAD ligation), significant differences in cardiomyocyte biology between the various mammalian mouse model species and humans (especially with regard to multinucleation and ploidy), a limited number of non-invasive assays, experiments, and imaging techniques, and the challenge obtaining healthy or ischemic neonatal human cardiac tissue to assess whether pathways with therapeutic promise identified in other mammals are present and function analogously in humans.

Although much research on cardiac regeneration has focused on the importance of cardiomyocyte renewal, cardiomyocytes comprise only a minority of the heart’s cells by number^12–14^. Cardiomyocyte renewal is one of the final steps in a coordinated and complex series of events involving many other cell types. Indeed, recent investigations have unearthed mechanisms by which non-cardiomyocytes, such as endothelial cells, fibroblasts, macrophages, and other immune cells, play critical roles in modulating the regenerative capacity of the injured heart. For example, in order for cardiomyocytes to repopulate the infarcted myocardium, first perfusion must be restored and cellular debris must be cleared for extracellular matrix (ECM) remodeling. The interdependency and spatiotemporal coordination of these processes during natural cardiomyocyte regeneration, as well as the degree to which each of these cell types regulates this process, remains incompletely understood. This review highlights the roles of these non-cardiomyocytes and their respective contributions to cardiac regeneration, with emphasis on natural heart regeneration after cardiac injury during the neonatal period.

## Mammalian response to cardiac injury

In adult mammals, ischemic injury to the myocardium is marked by widespread cell death^15^. The cellular response to ischemia follow a characteristic triphasic pattern^12,16^. The first phase involves influx of Ly-6C(hi) macrophages and neutrophils to generate a transient high-inflammatory state characterized by elevations in IL-1β, IL-6, and TNF-α^17^. This is followed by a second phase in which fibroblasts and resident endothelial cells are recruited via chemokine-secreting Ly-6C(lo) macrophages^12,18^. Transforming growth factor-β (TGF-β) is notable among these released factors for its ability to convert fibroblasts to myofibroblasts which induce ECM production and collagen deposition, constituting early scar formation^12,19,20^. In the third and final phase, the recruited macrophages undergo apoptosis, collagen fibers cross-link, and the mature scar is formed^12,21,22^. Often there is some temporal overlap between the three mechanistically unique phases^16^. Unfortunately, cardiomyocyte turnover in adult mammals proceeds extremely slowly, renewing at a rate of <1% annually for most of adult life, preventing meaningful natural cardiomyocyte regeneration after injury^23^. Although much has been done to elucidate mechanisms of cardiac regeneration in fish and amphibians, this review focuses on what is known in mammals.

Interestingly, neonatal mammals are transiently capable of natural heart regeneration following cardiac injury. In this review, natural heart regeneration refers to the heart’s intrinsic physiologic mechanism, naturally activated in the setting of injury, to regenerate cardiac structure and function in the absence of administering exogenous factors. Neonatal mice which undergo apical resection or ligation of the left coronary artery on postnatal day 1 (P1) exhibit complete cardiac regeneration with proliferation of cardiomyocytes and minimal fibrosis, although this ability is lost if injury occurs after postnatal day 7 (P7)^15,24^. An apparently conserved neonatal cardiac regeneration response has also been identified in other mammalian species as well, including rats^25^, rabbits^26^, and pigs^27,28^. In humans, cardiomyocyte turnover and renewal has been detected in the postnatal human heart^23,29,30^. One study has even shown functional restoration post-injury in a neonatal human heart^31^. In addition to preserving normal cardiac structure and function, natural heart regeneration in neonatal mammals has also been demonstrated to restore healthy epicardial conduction dynamics and preserve native left ventricular tissue biomechanics^32–34^.

**Fig. 1:**
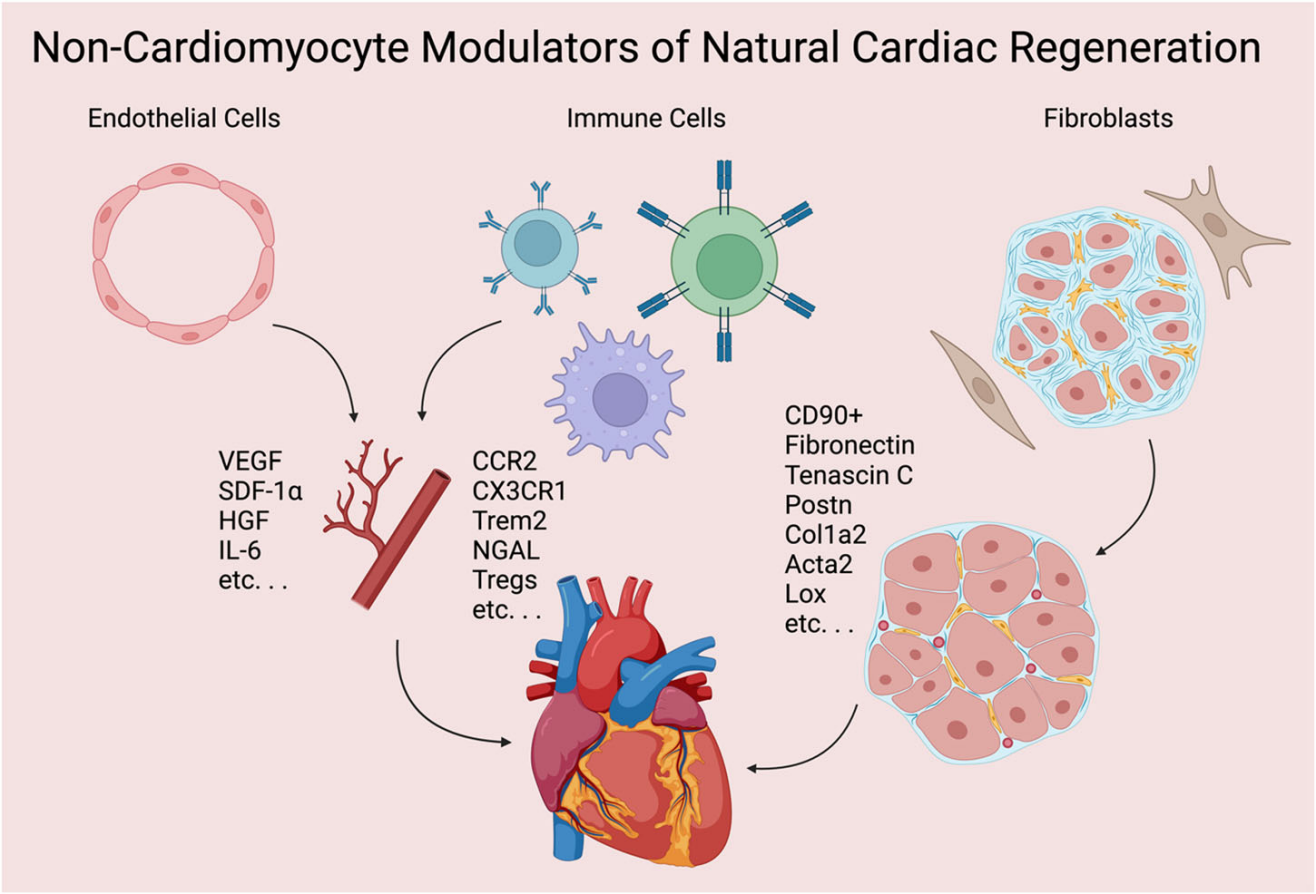
Non-cardiomyocyte modulators of natural cardiac regeneration. Created with BioRender.com.

Although cardiomyocyte proliferation plays a central role in the natural regeneration response and cardiomyocytes make up 70-85% of cardiac cells by volume, they comprise only 30-40% of cardiac cells by number^12^. The remaining 60–70% of cardiac cells, including endothelial cells, macrophages and other immune cells, and fibroblasts, are essential contributors to clearing debris, restoring structural integrity, and facilitating reperfusion to permit proliferation of new cardiomyocytes in natural regeneration (Fig. 1). The rest of this review will focus on these three cell types.

## Endothelial cells and pericytes

Endothelial cells make up the most abundant cardiac cell type by number, accounting for over 50% of cells in the mouse and human heart, and have been highly implicated in the cardiac regeneration response^35–37^. Following injury, revascularization of the injured myocardium via migration and proliferation of vascular endothelial cells from the surrounding tissue precedes cardiomyocyte regeneration^36–38^.

Endothelial cells have been implicated and targeted in studies of the transcriptional response that occurs following infarction in adult and neonatal mice. Quaife-Ryan et al. observed that vascular endothelial cells activated a unique and robust transcriptional response after infarction in adult mice that contrasted with the neonatal response to infarction^37^. In the 1 day-old neonate, the transcriptional activity of three specific cell groups (vascular endothelial cells, CD90+ fibroblasts, and cardiomyocytes) associated with Wnt signaling were highly upregulated compared to their 56 day-old adult counterparts^37^. Interestingly, Quaife-Ryan et al. noted that Wnt-associated genes were among three of the highest-ranked neonatal transcriptional networks by expression, the other two being E2f1 and Foxm1, which are involved in cell cycle progression and proliferation^37^. Furthermore, they found that neonatal vascular endothelial cells were enriched with cell cycle-associated transcription factors (an upregulation of pro-mitotic transcription factors), in contrast to their adult counterparts which were enriched with C3-complement-induced transcriptional networks (an upregulation of inflammatory factors)^37^.

Proangiogenic effects of endothelial cells also play a role in the mammalian response to cardiac injury. Stromal cell-derived factor 1 alpha (SDF-1α) is a potent endothelial cell progenitor stem cell chemokine that initiates neovascularization after injury^39^. With SDF-1α as a promising target, multiple groups have investigated this chemokine as a method of exogenously-induced angiogenesis in addition to restoring biomechanical properties. In 2011, Hiesinger et al. developed an engineered SDF-1α analog (known as ESA) which was shown to effectively stimulate microrevascularization in a mouse model of cardiac injury^39^. Angiogenesis after myocardial injury has been associated with improved biomechanics following infarct in male Wistar rats as shown by Wang et al. in 2019^40^. In their study, intramyocardial injection of ESA reduced infarct size, improved ventricular remodeling and function, and conserved native left ventricular biaxial mechanics^40^. Under hypoxic conditions, SDF-1α and other proangiogenic factors are upregulated including vascular endothelial growth factor (VEGF), hepatocyte growth factor (HGF), and IL-6, improving neovascularization and functional recovery and reducing infarct size^41–45^. SDF-1α has been shown to induce chemotaxis of endothelial progenitor stem cells to stimulate neovascularization and preserve cardiac functional capacity in ovine models of myocardial infarction (MI)^46^. These new capillaries resulted in significantly increased arteriolar perfusion, permitting ECM retention, reducing infarct expansion, and improving left ventricular function^46^. Following apical resection, endothelial cells have also been shown to populate the site of injury early after resection where they generate arteries preceding cardiomyocyte growth^47^. Ingason et al.’s findings suggested that after injury, arterial endothelial cell-derived SDF-1α may promote a sequence of cardiomyocyte migration following angiogenesis and arteriogenesis^47^. In 2018, Goldstone et al. observed that while SDF-1α exerted a proangiogenic effect after infarction, it did not change the proportion of circulating bone marrow-derived endothelial cells, suggesting that SDF-1α’s proangiogenic effect likely arises via formation of new blood vessels from pre-existing endothelial cells as opposed to bone marrow-associated vasculogenesis (de novo blood vessel formation)^48^.

In 2019, Das et al. identified a mechanism by which neonatal mouse hearts induced collateral artery reassembly following infarct^49^. They found that while it was indeed associated with endothelial cells it was distinct from previously established mechanisms of de novo arteriogenesis^49^. The previously described mechanisms of collateral artery formation were arterialization (in which capillaries convert to arteries) and arteriogenesis (in which existing collateral arteries are widened)^49–51^. However, in this novel mechanism (termed artery reassembly) single arterial endothelial cells travel and coalesce together to generate collateral arteries following coronary ligation^49^. In artery reassembly, these neonatal collateral arteries form from an arterial source and in places were collateral arteries were previously absent, distinguishing this pathway from arterialization and arteriogenesis, respectively^49^. In 2023, Das’ group identified that a key driver of the proliferation step of artery reassembly in neonatal mouse hearts post-injury is arterial VegfR2^52^. Their experiments demonstrated that arterial VegfR2 was downregulated in adult mouse hearts (a population in which artery reassembly does not occur after injury), and that collateral artery formation was rarely observed in arterial VegfR2-knockout models^52^.

While studies of revascularization after infarction have traditionally investigated pathways involving endothelial cells, the role of perivascular cells, called pericytes, remain enigmatic, in part due to the absence of unequivocal cell markers and tools for characterization and lineage tracing^53^. In healthy, homeostatic microvessels, pericytes and endothelial cells are separated by the vascular basement membrane and interact via peg-socket junctional complexes at fenestrations in the basement membrane to regulate the contractility of normal blood vessels^54–56^. Quijada et al. found in 2023 using multiple lineage-tracing mouse models that following myocardial infarction, pericytes traveled to the site of injury and expressed profibrotic genes^55^. When the same group genetically ablated Cspg4-expressing cells, a receptor downstream of the cardiac pericyte pathway they identified via single-cell RNA sequencing, they observed decreased cardiac function and increased mortality in the 2nd week following infarct^55^. Although comparably fewer studies have interrogated the downstream effects of pericyte activation, hyper-activation, under-activation, and deletion in the mammalian heart, early efforts have revealed promising mechanistic links between cardiac pericytes and the complex myocardial response to ischemic injury.

## Macrophages and immune cells

Macrophages are mononuclear phagocytes that act as part of the innate immune system to modulate inflammation via paracrine signaling. Following tissue damage, myocardial inflammation is initiated in part by the entry and activation of macrophages, monocytes, neutrophils, T cells, B cells, natural killer (NK) cells, and other immune cells into affected myocardium^57^. The macrophages then experience functional and morphological changes in their roles as mediators of tissue fibrosis and repair^57^. Macrophages attenuate myocardial injury through scavenging of cellular debris and through secretion of cytoprotective factors such as IL-10, myeloid-derived growth factor, and fibroblast growth factor-1, which act to inhibit proinflammatory cytokines and to reduce the fibrosis response^12,58–62^. Recent studies have suggested that waves of phenotypically distinct macrophages when temporospatially coordinated play a much greater and more nuanced role than previously appreciated in natural cardiac regeneration.

In 2007, Pittet’s group observed that two functionally distinct macrophage subgroups are recruited post-ischemic injury in mouse myocardium^63^. Functionally, the CCR2+ macrophage response is implicated in the digestion of damaged tissue and the CX3CR1+ macrophage response facilitates tissue healing via angiogenesis and collagen deposition^63^. In the early phase (phase I) following infarction, Ly-6C(hi) macrophages (which are most abundant in the early phase and have pro-inflammatory function) accumulated in wild-type and CX3CR1-/- mice but were nearly absent in CCR2-/- mice, suggesting early Ly-6C(hi) macrophage accumulation in phase I relies on CCR2 (a receptor for monocyte chemoattractant protein 1, or MCP-1) but does not depend on CX3CR1 (a receptor for fractalkine)^63^. Ly6C(lo) macrophages, which are most abundant in the later phase and are primarily angiogenic via expression of VEGF, were found in only small quantities across all three mouse lines throughout phase I^63^. In contrast, during the late phase (phase II) following infarction, Ly-6C(hi) macrophages were found in low quantities across wild-type, CCR2-/-, and CX3CR1-/- mice^63^. However, in phase II Ly-6C(lo) accumulated in wild-type and CCR2-/- mice but failed to accumulate efficiently in CX3CR1-/- mice, indicating that in phase II, Ly-6C(lo) macrophage accumulation relies on CX3CR1^63^.

Many studies elaborating on these findings have confirmed the importance of CX3CR1+ and CCR2+ macrophages in the healing response. A recent study by Vagnozzi et al. implicated these two macrophage subpopulations in improved heart function after ischemic injury without a corresponding increase in new cardiomyocyte production^64^. They found that an acute sterile immune response defined by temporal and regional induction of CCR2+ and CX3CR1+ macrophages within 8 weeks after injury improved heart function^64^. They also observed post-infarct mortality was significantly increased in CX3CR1-null mice, supporting the hypothesis that CX3CR1+ cells play a role in long-term pathophysiological remodeling via possible mechanisms such as influencing infarct maturation or the subsequent fibrotic response^64,65^. They conclude that the observed benefit to the infarcted region is based in the acute inflammation that occurs during the wound healing response, a mechanism characterized by temporary stimulation of the intrinsic wound healing cascade and macrophage subtypes^64^. Vagnozzi et al.’s findings support another recent paper from Epelman’s group which showed that inducible depletion of CX3CR1+ macrophages promoted pathological remodeling and increased mortality in mice, further supporting the role of these immune cells in restoration of cardiac function post-injury^65^. The Lavine group also recently found that tissue-resident CCR2+ macrophages promote monocyte recruitment through release of monocyte chemoattractant proteins (MCPs) with a myeloid differentiation primary response 88 (MYD88)-based mechanism as intermediary, whereas CCR2- macrophages act in opposition to inhibit recruitment of monocytes^65^. This is additive to previous studies which implicated direct CCR2 functional pathway inhibition in attenuation of left ventricular remodeling post-infarct^66^.

Although macrophages and their subpopulations (including the previously mentioned CCR2+ and CX3CR1+ populations) are essential to the regenerative response, Aurora et al. demonstrated in the neonatal mouse heart that while macrophages are not directly required for cardiomyocyte proliferation, total regeneration cannot occur without neovascularization which is dependent on macrophages, suggesting that cardiomyocyte proliferation is only one component of the functional regenerative response^67^. When they removed approximately half of the mononuclear phagocyte (CD11b^+^Ly-6G^–^) population in P1 neonates via clodronate liposome-mediated depletion, neovascularization deficiencies were incurred, implicating P1 macrophages in the neovascularization response post-injury^67^. In 2022, the Chung group showed that the macrophage subset Trem2(hi) is upregulated in the late stage after MI in adult mice, and that injection of soluble Trem2 resulted in improved cardiac function post-infarct in a similar mouse model^68^. In concert, these studies imply that macrophages and their subpopulations play essential roles in the regeneration process after injury, and their manipulation may provide experimental means by which therapies which strive to induce regeneration may be refined.

Other immune cell mechanisms may influence the degree of natural heart regeneration as well. A recent study found that T cell development (or transfer of adult IFN-γ-producing T-cells) impaired cardiac regeneration in neonates and was associated with greater functional and structural cardiac damage, revealing a “trade-off” relationship between cardiac regenerative potential and T cell development in neonatal mice^69^. T lymphocytes are largely made up of CD4+ and CD8 + T cells^70^. Ordinarily, CD4 + T cells activate the innate immune system, cytotoxic T cells, B-lymphocytes, non-immune cells, and include many subset cell populations with distinct roles in the natural immune response^70^. In non-regenerative P8 mice, ablation of CD4 + T cells was shown to increase regeneration after infarct, and neonatal mice reconstituted with adult T cells were shown to have a reduced regenerative response to alterations in the adaptive immune system^71–73^.

Regulatory T cells (Tregs), a subset of T cells that prohibit autoimmunity and maintain peripheral tolerance, have also been shown to promote cardiomyocyte proliferation in a paracrine manner^74–76^. Increasing Tregs via CD28 superagonist administration or via adoptive transfer reduced infarction-associated ventricular remodeling in mice and rats^74,77,78^. Specifically, Li et al. showed that the potentiation of neonatal cardiomyocyte proliferation is mediated by factors secreted from Tregs, specifically CCL24, GAS6, and AREG^74^. In addition, myocardial Tregs express Sparc (secreted acidic cysteine-rich glycoprotein, also called osteonectin, a matricellular protein that binds collagen), which has been shown in mouse models to increase collagen and boost infarct zone maturation resulting in a net cardioprotective effect following infarction^79^. The Treg results, in concert with the inverse relationship between immune maturity and regeneration, have several intriguing implications that warrant investigation. These include the potential role of immunosuppression or rheumatologic therapy in cardiac regenerative therapies, incorporating organisms with varying degrees of immune competence into experiments, and reproducing previous studies to interrogate whether better outcomes correlate inversely with immune function or activation.

Neutrophils also play a key role in the modulation of cardiac regeneration. The reparative role of neutrophils (which migrate in large proportions to the infarct site in the first few hours of ischemia) is a new and much-debated phenomenon, given that they have traditionally been associated only with pro-inflammatory damage to myocardial tissue^80^. The Steffens group found that following infarction, neutrophil-depleted mice had higher levels of fibrosis, decreased cardiac function, and higher likelihood of developing progressive heart failure^81^. They identified that a key mediator of this process was neutrophil gelatinase-associated lipocalin (NGAL) which induces macrophages with a high capacity for scavenging apoptotic cells^81^. When the same group restored NGAL via injection in neutrophil-depleted mice, the macrophage phenotype that promoted recovery was restored, suggesting that following infarction neutrophils polarize macrophages toward a phenotype with high ability to remove apoptotic cells, thereby improving the healing response^81^. Inadequate removal of apoptotic cells following MI results in adverse remodeling, delays inflammation resolution, and reduces cardiac function^81,82^. In addition, phagocytic clearance of necrotic myocardium and cellular debris by neutrophils promotes repair^80,83^.

These studies suggest that the inflammatory microenvironment generated by neutrophils may play a crucial role in the recruitment of cardiac progenitor cells^84^. They also highlight interesting questions of future experiments incorporating neutrophil supplementation into models of cardiac regeneration to investigate their effects on regenerative outcomes. Interrogating the relationship between selective immune cell supplementation, the use of immunosuppressive therapy, and possible interactive effects between them on mammalian models of cardiac regeneration may elucidate means by which current cardiac regenerative therapies may be improved.

## Fibroblasts

Fibroblasts play a key structural role in the healthy heart and in the natural response to cardiac injury by depositing and maintaining ECM^85^. In healthy homeostatic myocardium, cardiac fibroblasts are considered ‘quiescent’ cells without high proliferation or ECM turnover^86,87^, and in adult mouse hearts CD90+ fibroblasts (the key cell type involved in fibrosis, inflammation, and cell proliferation and differentiation) comprise ~30% of nonmyocytes^37,88,89^. The ECM is an essential part of healthy myocardium, is comprised of a complex interconnection of components, most common among them collagens I, III, and IV, serum albumins, proteoglycans, elastins, and glycosaminoglycans, and acts as a repository for anchored growth factors, chemokines, cytokines, proteases, protease inhibitors, and noncoding RNAs^90^.

However, in response to hypoxia or injury, cardiac fibroblasts activate to increase synthesis of ECM constituents^86,91^. Excessive structural remodeling post-injury via activation of cardiac fibroblasts and the accumulation of ECM is pathophysiologically linked to scar formation, myocardial stiffening and decreased cardiac contractility^92,93^. Modulation of these remodeling pathways may provide a means by which natural heart regeneration is at least partly facilitated, perhaps by establishing a conducive matrix for revascularization and eventual cardiomyocyte migration.

Neonatal mouse hearts retain a robust regenerative response facilitated by inflammation and cardiomyocyte proliferation without extensive fibrosis^24,86,94^. Transcriptomic and phenotypic analysis of fetal human cardiac fibroblasts revealed smaller size and increased cell turnover when compared to adult human fibroblasts^86,95^. Transcriptomic analysis in mice also showed that compared to adult mice, embryonic mouse cardiac fibroblasts displayed higher expression of fibronectin (FN), tenascin C (TNC), collagen genes, and Postn, an expression profile associated with increased cardiomyocyte proliferation^86,96^. This expression profile points to fibroblasts as one of several important regulators of cardiac regeneration, though the relative contributions of fibroblasts, especially their role in the tenuous balance between sufficient fibrosis to prevent tissue from becoming aneurysmal and excessive fibrosis in the post-infarct period, remains an area of active study^86,96^. FN, TNC, and collagens are components of the mammalian cardiac ECM^97^. Postn is a protein involved in wound healing and is expressed following injury to activated fibroblasts^86,98^. In adult mice, post-injury ablation of Postn+ activated fibroblasts reduced fibrosis, increased cardiomyocyte abundance in the scar area, and improved cardiac function^86,99^. Despite the apparent cardioprotective effect of ablating Postn+ activated fibroblasts, cells expressing Postn have also been shown to be essential to the cardiac healing process following infarction, highlighting the role of Postn in cardiac regeneration as an active and incompletely understood area of research^100^. When Shimazaki et al. generated a mouse model of Postn knockout, they observed that after myocardial infarction there was impaired collagen fibril formation resulting in cardiac rupture, a phenotype that was rescued via gene transfer of a spliced version of Postn^100^. Experimentally manipulating the quantity of these ECM components using gene knockout or overexpression techniques in models of cardiac regeneration in order to assess their individual contributions to cardiomyocyte proliferation can highlight factors whose use may enhance cardiac function in current regenerative therapies.

Biomechanically, the stiffness of the ECM that forms after infarct has been shown to be inversely proportional to cardiac regenerative capacity in neonatal mice, suggesting that relative ECM composition also contributes to the regeneration response^86,101,102^. Increased ECM stiffness has also been proposed to inhibit the cardiomyocyte cell-cycle^86,103^. Cross-linking of ECM components is also an important regulator of stiffness in vivo^101^. Cardiomyocyte proliferation in adult mammals is also markedly lower than in neonates, and the fibrotic scar that forms and matures in absence of subsequent replacement by cardiomyocytes has been shown to pathologically reduce cardiac function and compliance^86^. Notably, between P1 and P2 mice, Notari et al. found that stiffness of local microenvironment is a crucial regulator of regenerative capacity even within the neonatal conserved window, observing that decreasing local ECM stiffness was associated with increased regeneration after injury^101^. A cellular-level explanation for this difference can be found in the Tzahor group’s paper, which demonstrates that in stiffer microenvironments, proliferating cardiomyocytes beget binucleated cardiomyocytes, in contrast to normal environments where two mononucleated cells are generated via cytokinesis from each proliferating cardiomyocyte^86,102^.

In 2017 transcriptomic analysis also uncovered an unexpected finding regarding the role of fibroblasts in natural regeneration. Quaife-Ryan et al. found that in adult mice, CD90+ fibroblasts retained a “transcriptional plasticity” which allowed them to regress to a “neonatal-like” state after infarction, so named because the state bears closer resemblance to neonatal cells (healthy or infarcted) than healthy adult cells^37^. However, they observed that adult cardiomyocytes failed to revert to neonatal transcriptional networks (especially ones implicated in cell cycle regulation), contrasting with previous dogma suggesting neonatal transcriptional reversion occurred during cardiac hypertrophy or failure in adults, and they hypothesized that reversion may be “an exception rather than the rule”^37^. These findings are consistent with the hypothesis that fibroblast activity and ECM deposition modulate cardiac regeneration, but the exact mechanism or mechanisms by which this occurs remains a fertile opportunity for further investigation.

The role of cardiac fibroblasts is not only limited to supporting cardiomyocyte proliferation. The Molkentin group suggested that cardiac fibroblasts are modulated by macrophage subgroups to influence restoration of mechanical function, demonstrating via gene expression analysis that isolated CCR2+ macrophages increased fibroblast expression of collagen type I α 2 (Col1a2), smooth muscle α-actin (Acta2), and lysyl oxidase (Lox)^64^. The expression of these three genes was reduced modestly by isolated CX3CR1+ macrophages^64^. However, CX3CR1+ macrophages increased connective tissue growth factor (Ctgf) expression in fibroblasts, suggesting that macrophage subgroup influences infarct structure via modulation of cardiac fibroblasts^64^.

## Future directions

Cardiomyocyte proliferation is the central process in natural cardiac regeneration. Ultimately, cardiac regeneration depends on the generation of new cardiomyocytes and non-cardiomyocytes are likely to influence regeneration via adjunctive yet critical roles. However, in the complex crucible of cardiac regeneration, the modulation of these cardiomyocytes in addition to the modulation of functional and structural endpoints consistent with restoration of cardiac function post-injury are facilitated by a host of other cell types such as endothelial cells, macrophages, fibroblasts, T cells, and more. Given the complex interconnection of cardiac cell lines in the natural regeneration response and recent findings of improved functional restoration post-infarct in animal models by means of non-cardiomyocyte modulation of the regeneration response, future therapies may integrate our understanding of these non-cardiomyocyte cell types as new avenues to maximize functional cardiac recovery following ischemic injury.

### Reporting summary

Further information on research design is available in the Nature Research Reporting Summary linked to this article.

### Supplementary information

Reporting Summary

## Data Availability

No new data were created in the writing of this review article.
